# Erratum to: Superoxide dismutase and catalase: tissue activities and relation with age in the long-lived species *Margaritifera margaritifera*

**DOI:** 10.1186/s40659-016-0096-1

**Published:** 2016-08-25

**Authors:** Carlos Fernández, Eloísa Ferreira, Eduardo San Miguel, Almudena Fernández-Briera

**Affiliations:** 1 Department of Genetics, Faculty of Veterinary, University of Santiago de Compostela, Lugo, Spain; 2 Department of Biochemistry, Genetics and Immunology, Faculty of Biology, University of Vigo, Vigo, Spain

## Erratum to: Biol Res 42: 57–68, 2009 DOI 10.4067/S0716-97602009000100006

Unfortunately, the original version of this article [1] contained an error. The name of one of the authors, Eloísa Ferreira, was not included in the author list.

The correct author list and the new “Authors’ contributions” section have been correctly included in this erratum.
